# Disentangling the relationship between environmental drivers and productivity in subalpine wet grasslands

**DOI:** 10.3389/fpls.2025.1582124

**Published:** 2025-06-03

**Authors:** Shuqiao Zhang, Langlang Shu, Wendou Liu, Zizhi Wang, Wengui Wu, Shengxi Liao

**Affiliations:** ^1^ Institute of Highland Forest Science, Chinese Academy of Forestry, Kunming, Yunnan, China; ^2^ School of Agriculture, Food and Ecosystem Sciences, Faculty of Science, The University of Melbourne, Creswick, VIC, Australia; ^3^ Research Institute of Forestry Policy and Information, Chinese Academy of Forestry, Beijing, China

**Keywords:** biodiversity, biomass, multivariate modeling, structural equation modeling, plant ecology, vegetation

## Abstract

**Introduction:**

Grasslands exhibit significant variability in productivity across fine spatial scales, which is crucial for understanding terrestrial carbon cycling, particularly under global climate change. While alpine grasslands have been extensively studied, subalpine wet grasslands (2000–4000 m) remain underexplored. Investigating their productivity and responses to environmental factors is essential for a comprehensive understanding of ecosystem dynamics in these regions.

**Method:**

We applied destructive sampling techniques, optimized grassland investigation, and employed multivariate modeling to examine how different environmental variables influence grassland biomass. An 80-plot field-based dataset was established in a subalpine wet grassland.

**Results:**

Our findings reveal that plant biomass peaked at elevations between 3400 and 3500 m. Belowground biomass accounted for 85% of total productivity, with the majority contributed by dominant species. Vegetation-related variables, such as coverage and root/shoot ratio, were the primary determinants of aboveground biomass, whereas soil properties were key regulators of belowground biomass. Although direct and indirect effects of landform and climatic factors influenced total biomass, the patterns of total and belowground biomass were consistent. The results underscore the significant positive impact of vegetation cover, root-to-shoot ratio, and soil conditions on grassland productivity. Notably, soil organic carbon, water content, and the nitrogen-to-phosphorus ratio affected belowground biomass.

**Discussion:**

These insights enhance our understanding of the intricate interactions between climate, soil, landform, and plant communities in influencing grassland biomass and highlight the importance of preserving plant diversity and maintaining optimal soil conditions in subalpine wet grasslands. One grassland does not fit all; fine-scale classification is essential to capture the variability in productivity across different grassland types.

## 1 Introduction

Grasslands cover approximately 30~40% of the Earth’s terrestrial surface and store up to 20% of the carbon pool, which constitutes one of the most extensive biomes worldwide (White et al., 2000; Bardgett et al., 2021; Lewińska et al., 2023; Thébault et al., 2014). Grassland ecosystems are integral to numerous terrestrial processes, including climate regulation and soil and water conservation, providing critical habitat and food sources for herbivores (Bengtsson et al., 2019). Despite their ecological significance, many grassland systems have become vulnerable due to heavy grazing compounded by insufficient conservation measures. This vulnerability is particularly pronounced in mountainous meadows, at the timberline ecotone, where environmental conditions grow increasingly severe.; thus, these areas are more susceptible to climate change (Beniston, 2003). Therefore, understanding how environmental factors influence grassland ecosystem health in these ecosystems is crucial.

Plant biomass is an important indicator of ecosystem productivity. It is influenced by biotic and abiotic environmental conditions and is crucial in the carbon cycle and carbon stocks (Hu et al., 2018). Accurate biomass estimation (especially belowground) is required to understand the terrestrial carbon cycling dynamics (Yang et al., 2008). Climate has a significant effect on biomass in montane grasslands. Temperature and precipitation patterns affect plant growth and productivity (Körner, 2021). Cooler temperatures and higher precipitation in high-altitude environments generally slow down plant metabolic rates and decrease productivity (Volk et al., 2021). Irregular or extreme weather events can disrupt plant development and reduce productivity (Liu et al., 2021a). In addition, the regional climate can affect the spatial pattern of community biomass (Bai et al., 2008). Grace et al. (2016) stated that the combined effects of large gradients of temperature and precipitation could be the reason for the heterogeneity in aboveground biomass. On a regional scale, precipitation and temperature may affect vegetation growth and distribution in a consistent predictive relationship, influencing biomass. Topographic variables, such as slope and aspect, affect biomass and plant communities by influencing regional temperature and moisture availability (Fang et al., 2005). For example, precipitation usually increases plant productivity, but the effect of precipitation on wet grassland productivity decreases with increasing elevation (Xun et al., 2024). A study reveals that elevation may negatively impact aboveground biomass in alpine grasslands (Bhandari and Zhang, 2019); however, the effect of elevation on plant biomass in subalpine wet grasslands remains unclear.

Soil properties impact plant biomass, and soil physical and chemical properties affect plant species richness and community biomass by influencing soil water holding capacity, mobility (Schoonover and Crim, 2015), soil fertility (Tiessen, 1994), and acidity (Palpurina et al., 2017). Soil nutrients contribute to carbon uptake, increasing plant growth and biomass (Lee et al., 2013). Landform characteristics can be relatively uniform at large spatial scales, but microtopographic conditions at small scales can enhance soil microbial activity (Marshall et al., 2014), indirectly affecting plant biomass. Biotic factors, such as species composition and diversity, impact biomass through competitive dynamics and mutualistic interactions. The root/shoot ratio has been used to calibrate and estimate C stocks (Tilman et al., 1997) to perform terrestrial ecosystem carbon modeling (Thuynsma et al., 2014). Species richness and inter/intra-specific competition are also correlated with grass biomass (del-Val and Crawley, 2005; Partzsch, 2019).

Although many studies have shown that environmental conditions (biotic and abiotic) influence plant biomass (McCarthy and Enquist, 2007), little is known about the interaction of these environmental drivers on plant biomass (Bello et al., 2013). The effects of environmental factors on biomass vary, and many scholars have focused on the effects of a single factor (Jones, 2014; Raich et al., 2014; Yang et al., 2016). Since many environmental factors and experimental conditions impact plant biomass, more in-depth research is required to assess the relationship between environmental drivers and plant biomass. Many studies have focused on broad-scale patterns (Yang et al., 2010) or specific species (Dorner et al., 2024). However, mechanistic understanding of how fine- to meso-scale environmental interactions modulate biomass distribution in these ecosystems remains limited. Additionally, understanding the drivers is essential because high-elevation grasslands are highly sensitive to climatic fluctuations and anthropogenic pressures (Liu et al., 2021a). Grassland studies have been conducted predominantly in alpine (>4000 m) (Genxu et al., 2008; Wang et al., 2012) and low-elevation (<2000 m) areas (Hooper and Johnson, 1999; Knapp et al., 2002), whereas few focused on subalpine grasslands at 2000–4000 m elevations.

Wet grasslands are ecosystems characterized by consistently high soil moisture levels and vegetation types adapted to such conditions, ranging from saturated to seasonally waterlogged areas. They occur in regions with a high water table or periodic inundation (Joyce et al., 2016). Abundant water sources, high biodiversity, and fertile soils make this ecosystem productive and worth studying. Since most studies defined alpine grasslands as areas above 4000 m (Genxu et al., 2008; Wang et al., 2012), we used the term subalpine grasslands to define grassland areas with elevations from 2000 to 4000 m near the timberline. Subalpine wet grasslands are located at the lower elevations of alpine regions and are characterized by high soil moisture and vegetation communities adapted to the alpine conditions. These grasslands occur between the montane and alpine zones and are influenced by high precipitation, cool temperatures, and relatively short growing seasons (Körner, 2021).

The synergistic effects of vegetation traits, edaphic factors, topographic variation, and climatic influences on productivity remain poorly resolved in subalpine wet grasslands. We posit that environmental variables exert deterministic control over biomass allocation in subalpine wet grasslands. The factors we analyze in this study include those considered in the above literature and understudied determinants with potential theoretical or statistical values, such as site locations, soil pH, and density. A thorough understanding of the relationships between environmental factors and biomass is crucial for predicting the response of these grasslands to environmental changes and devising effective management strategies to maintain their ecological integrity. Hence, this research aims to disentangle the complex relationships between plant biomass in subalpine wet grasslands and environmental factors, such as vegetation, topography, climate, location, and soil properties. The objectives are to (1) estimate the productivity in the study area, (2) analyze the correlations between aboveground, belowground, dominant species, and total biomass, (3) identify statistically significant environmental factors affecting biomass, and (4) analyze relationships and contributions of dominant factors to aboveground, belowground, and total biomass.

## 2 Materials and methods

### 2.1 Study area

**Figure 1:**
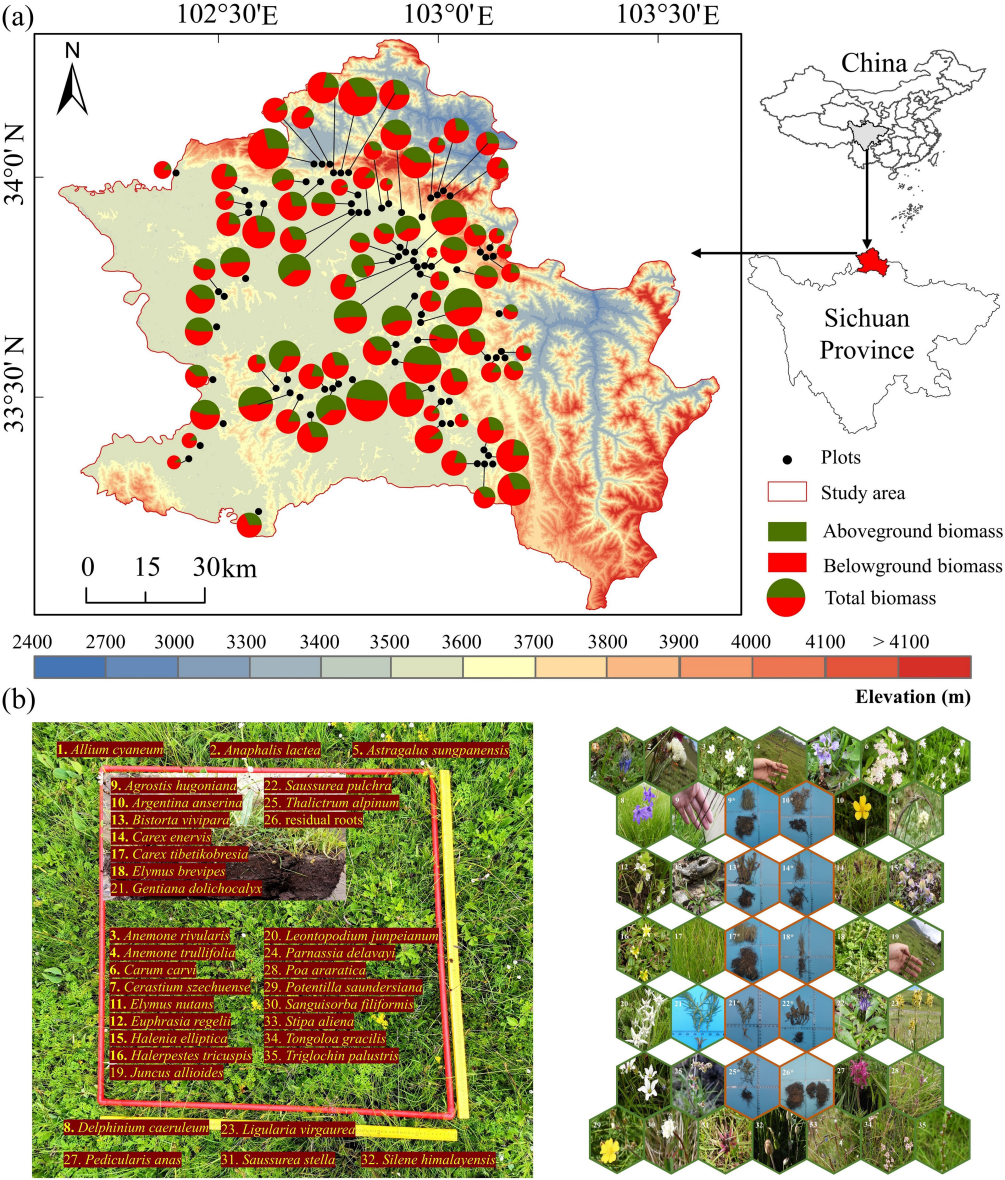
**(a)** Map of the study area (Zoige region) and elevation. **(b)** An example plot with names and photos of plant species. Fresh samples are shown in green frames, and lab-dried samples are shown in brown frames with “*” denoted after photo numbers.

The study area is located in the eastern Qinghai-Tibet Plateau. It covers an area of 10,164 km^2^ and has an average altitude of 3500 m (**Figure 1a**). We used the Sichuan Province Grassland Resource Survey Map (1:2.5 million scale), the Zoige County Grassland Type Map (1:700,000 scale), and grassland classifications used in recent Zoige studies (Bai et al., 2013; Liu et al., 2020) to choose six grassland types: marsh wetland, marsh grassland, wet grassland, subalpine wet grassland, subalpine shrub grassland, and alpine grassland. Due to the potential impacts of elevation on grassland biomass (Wang et al., 2024; Xun et al., 2024), we distributed 80 plots uniformly in wet grassland in different elevation zones. The study area (Zoige region) has diverse topographic conditions, including hills, plateaus, and valleys. The hilly plateau accounts for 69% of the total area. The study region has a wet, high-elevation, cold temperate climate. The mean annual precipitation ranges from 600 to 800 mm, and most precipitation falls in the rainy season (May to October). The mean annual temperature is 0.6 to 2 °C, and the annual temperature difference is 20 to 21 °C. The hottest month (June) has an average temperature of 10.8 to 12.6 °C, whereas the coldest month (January) has an average temperature of -10.2 to -7.2 °C. The average annual sunshine hours are 2000 to 2400 h. The soil types are diverse, but swamp soil and subalpine meadow soil are dominant, accounting for 33.1% and 30.15% of the total area, respectively (Wang et al., 2016). The unique geographical and climate conditions of this region lead to rich and diverse grassland resources. The grassland area is 8084 km^2^ (72.09% of the study area), and the major grassland types include alpine meadows and alpine semi-swampy areas. Dominant plant species include *Blysmus sinocompressus*, *Carex setschwanensis*, *Carex enervis*, *Carex tibetikobresia*, *Elymus nutans*, *Carex moorcroftii*, and *Carex muliensis*.

### 2.2 Experimental design and data collection

A field survey was conducted in August 2020 at peak biomass in areas of high rainfall (Hagiwara et al., 2010; Liu et al., 2021b). We used a 1×1 m plot for sample collection (**Figure 1b** is an example). The 80 sample plots were randomly selected in different elevation zones, considering accessibility (3200–3900 m) (**Figure 1a**). The minimum linear distance between the sample plots was 200 m. The dominant species and biomass of the plant community differed substantially at different sites. We chose undisturbed sites with stable species composition and biomass as sampling areas, representing more than 60% of the landform types in the local area. Water areas were not included in the survey.

The coordinates of the plots were recorded using a portable GPS unit, and topographic information, including elevation, slope, and aspect, was collected. We investigated plant attributes, including the height, dominant species, richness, and coverage in each plot. We used destructive sampling for vegetation analysis (Christensen and Hörnfeldt, 2003; Gonzalez-Cascon and Martin, 2018). All plant species in the plots were photographed, harvested, and cleaned with fresh water in the field. The samples were separated into individual species, which were identified in the lab. The soil was broken apart in a water-filled tray, and roots were removed manually with tweezers. The roots were separated into live (white, flexible) and dead (dark, brittle) parts, identified by morphology, and sorted by plant species before weighing. Unrecognized and broken roots were categorized as residual roots (**Figure 1b**-26). The fresh aboveground and belowground plant components were weighed separately using an analytical balance to determine the biomass (Liu et al., 2021b). The plant samples were placed into envelopes, labeled, and mailed to the laboratory to check the accuracy of the names. They were oven-dried to a constant weight at 65 °C for two days, and their dry weight was recorded to the nearest milligram (Hamrouni-Sellami et al., 2013).

Soil samples were collected at a depth of 30 cm using a soil corer. Four soil cores (100 cm^3^) were obtained in each plot and stored in labeled containers. Two cores were oven-dried to determine the soil bulk density and soil water content. The plant roots and gravel were separated from the remaining soil samples, and the material was air-dried and passed through a 2 mm sieve. The samples were used to determine soil physical and chemical properties, including soil organic carbon, soil total nitrogen, total phosphorus, and pH. Soil organic carbon was measured using the K_2_Cr_2_O_7_-H_2_SO_4_ oxidation method, and soil total nitrogen was measured using the Kjeldahl method. Soil total phosphorus was measured using the molybdenum antimony colorimetric method. Soil pH was obtained with a calibrated pH meter after 30 min of extraction in distilled water (soil: water ratio of 1:2.5) (Li et al., 2021). Mean annual temperature, mean annual precipitation, and solar radiation data were derived from WorldClim data (Fick and Hijmans, 2017). Landform data, such as slope and aspect, were extracted from a 12.5 m resolution digital elevation model obtained from the Advanced Land Observing Satellite (ALOS).

### 2.3 Analysis

We modeled 18 environmental variables (**Table 1**) that are known to have a significant effect on grassland productivity (Sun et al., 2021). We conducted Pearson correlation analysis to determine variables with a high correlation with aboveground, belowground, and total biomass and established a polynomial (quadratic) regression model to analyze the effect of the factors on biomass. The explanatory power of the model was assessed based on the significance (*P*-value) and the coefficient of determination (*R* ^2^). Principal component analysis (PCA) was used for dimensionality reduction to transform multiple indicators affecting the biomass of grassland vegetation communities into several composite indicators. The principal components reflect most of the information of the original multiple indicator variables (Marsboom et al., 2018). Stepwise regression analysis (SRA) was used to remove multicollinear variables (Ray-Mukherjee et al., 2014). The PCA and SRA results were combined to identify the critical factors affecting grassland biomass. Results from the literature and field observations were used to select key variables to minimize type I errors.

**Table 1:** Descriptive statistics and abbreviations of the 18 environmental factors used in this study.

Category	Variable	Abbreviations	Min	Max	Mean	SD
Vegetation	Species richness	SR	5	48	20	8.76
	Coverage (%)	Cover	0.3	1.0	0.8	0.22
	Root-shoot ratio	R/S	0.5	38.2	10	6.43
Soil	Soil organic carbon (g kg^-1^)	SOC	33.7	405.4	122.3	71.05
	Soil water content (%)	SWC	15.8	473.5	85.7	83.05
	Soil bulk density (g cm^-3^)	SBD	0.2	1.9	0.9	0.39
	Soil total nitrogen (g kg^-1^)	TN	1.5	18.2	6.5	3.57
	Soil total phosphorus (g kg^-1^)	TP	0.3	1.7	0.8	0.27
	Soil pH	PH	5.7	8	6.7	0.46
Landform	Elevation (m)	ELE	3320	3918	3482.7	109.06
	Slope (m/100m)	Slope	0	26.7	6.5	7.09
	Aspect (°)	Aspect	0	353.7	184.3	98.27
	Longitude (°)	LON	102.4	103.1	102.9	0.20
	Latitude (°)	LAT	33.2	34	33.7	0.22
Climate	Mean annual temperature (°C)	MAT	-0.2	2.5	1.7	0.63
	Mean annual precipitation (mm)	MAP	653	699	670.9	12.07
	Solar radiation (MJ m^-2^yr^-1^)	SOL	8009	8510	8211	115.53

The interactions between the driving factors and plant biomass were analyzed and plotted using a structural equation model (SEM) (Bollen, 1989). We used the SEM to evaluate the direct, indirect, and total relationships among the factors to quantify correlations and the variance instead of assuming that predictors were independent. We observed collinearity between the 6 soil property indices. PCA was used to reduce the dimensions of the soil property indices. The first two principal components were chosen to represent the soil properties. Standardization was performed to standardize the units. We used the LAVAAN package in R 4.1.2 (R Core Team) to implement the SEM, which is robust to multivariate non-normality (Niu and Ban, 2012). Model fitness was measured using the chi-squared test (P>0.05), comparative fit index (CFI>0.95), goodness-of-fit index (GFI>0.95), standardized root mean square residual (SRMR<0.06) and root mean square error of approximation (RMSEA<0.05). Variance Inflation Factors (VIFs) were used to quantify the degree of linear correlation between biomass and explanatory variables in the SEM. All statistical analyses were performed using IBM SPSS version 24.0 and R version 4.1.2, and the data were analyzed using SPSS v25 and OriginPro 2017 (IBM Corp).

## 3 Results

### 3.1 Biomass estimation and biotic factors

**Figure 2:**
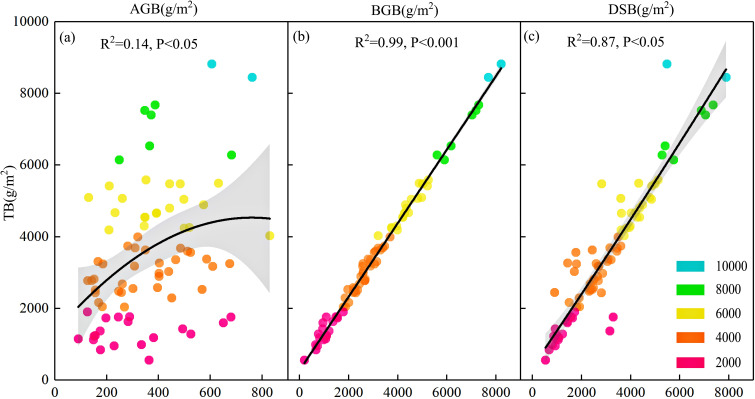
Relationships between total biomass and **(a)** aboveground biomass, **(b)** belowground biomass, and **(c)** dominant species biomass in subalpine wet grasslands. Solid lines represent least squares regression lines; shaded areas indicate 95% confidence intervals. Each point corresponds to a plot, with color gradients reflecting total biomass levels.

**Table 2** lists the descriptive statistics of the grassland biomass parameters. The average aboveground biomass, belowground biomass, total biomass, and dominant species biomass were 360.61 g/m^2^, 3126.65 g/m^2^, 3487.26 g/m^2^, and 3027.50 g/m^2^, respectively. Although most of the biomass occurred from 3400 to 3500 m, the biomass varied substantially in different plots, and the coefficient of variation ranged from 47% to 65%. Aboveground, belowground, and dominant species biomass showed a significant positive correlation with total biomass (**Figures 2a–c**). Belowground biomass was nearly nine times greater than aboveground biomass (**Table 2**). Belowground biomass and dominant species biomass were highly correlated with total biomass, with a degree of fit of 99% (P<0.001) and 87% (P<0.05), respectively (**Figures 2b, c**). The roots represented a large proportion of the total biomass. The root/shoot ratio ranged from 0.53 to 38.19, indicating high spatial heterogeneity. The average root/shoot ratio (10.10) is typically higher in subalpine wet grasslands than in temperate grasslands globally (root/shoot ratio of 4.2) (Mokany et al., 2006) and is comparable to that of the wetland arctic tundra ecosystem (root/shoot ratio of 11).

**Table 2:** Descriptive statistics of plant biomass parameters.

Variables	Abbreviations	Min	Max	Mean	SD	CV
Aboveground biomass (g m^-2^)	AGB	90.51	829.58	360.61	169.72	47.06
Belowground biomass (g m^-2^)	BGB	193.75	8213.12	3126.65	1797.73	57.50
Total biomass (g m^-2^)	TB	557.42	8818.96	3487.26	1851.81	51.10
Dominant species biomass (g m^-2^)	DSB	531.89	7881.71	3027.50	1646.11	64.28

### 3.2 Environmental factors

**Figure 3:**
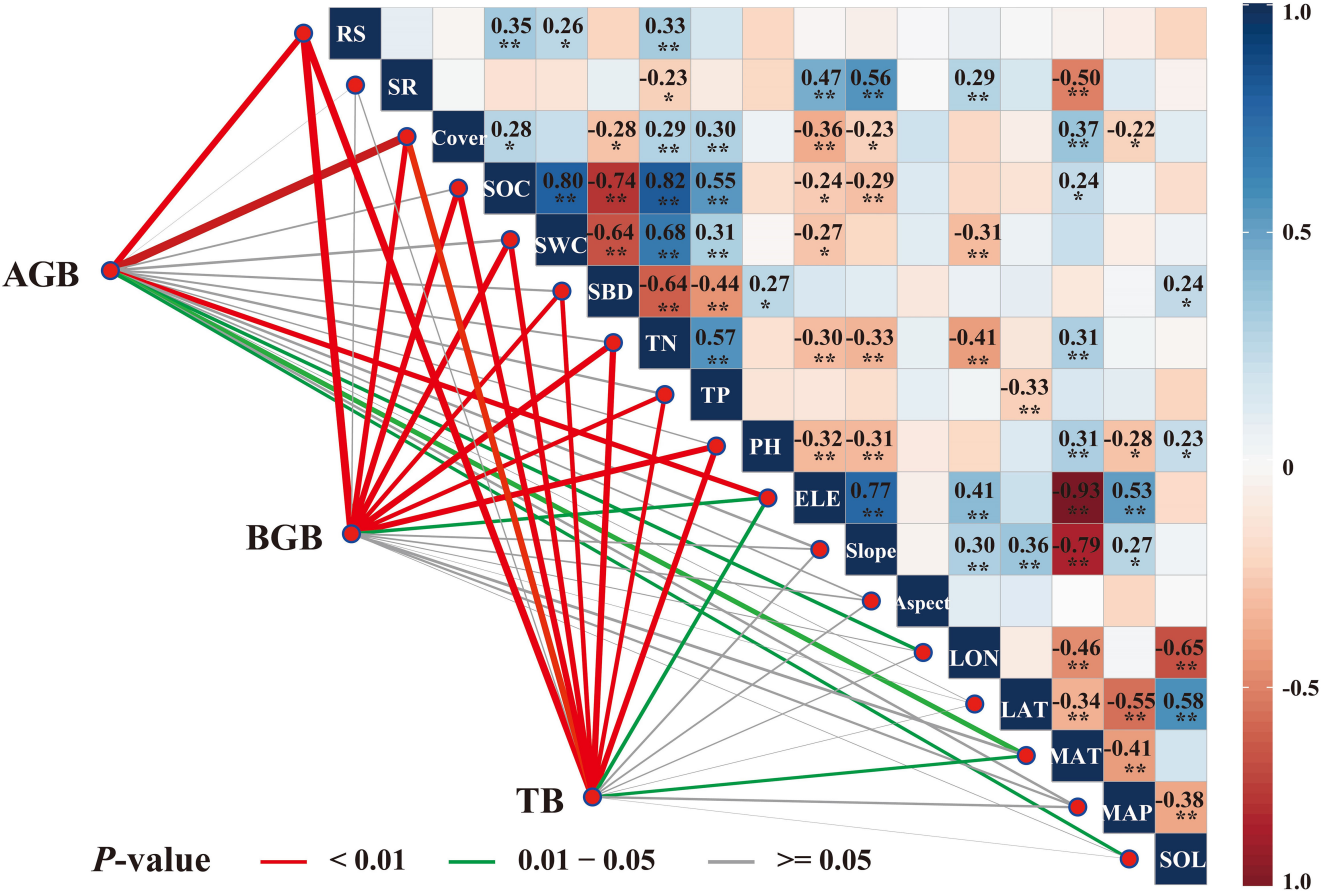
Pearson’s correlation coefficients (r) between biomass and environmental driving factors represented by color gradients and numerical values with asterisks, indicating both the strength of the correlation and its statistical significance. The significance levels are *P<0.05, **P<0.01, ***P<0.001.

We analyzed the correlations between plant biomass and the climate, topographic, vegetation, soil, and location factors (**Figure 3**). Aboveground biomass was highly correlated with the root-shoot ratio, vegetation cover, longitude, elevation, mean annual temperature, and solar radiation (*P*<0.05). Belowground biomass was highly correlated with the root-shoot ratio, vegetation cover, elevation, soil organic carbon, soil water content, soil bulk density, soil total nitrogen, soil total phosphorus, and pH (*P*<0.01). Likewise, total biomass was highly correlated with the root-shoot ratio, vegetation cover, elevation, mean annual temperature, soil organic carbon, soil water content, soil bulk density, soil total nitrogen, soil total phosphorus, and pH (*P*<0.05). Moreover, species richness, latitude, slope, aspect, and mean annual precipitation were not correlated with aboveground, belowground, and total biomass (*P*>0.05).

### 3.3 Relationships between biomass and key factors

**Figure 4:**
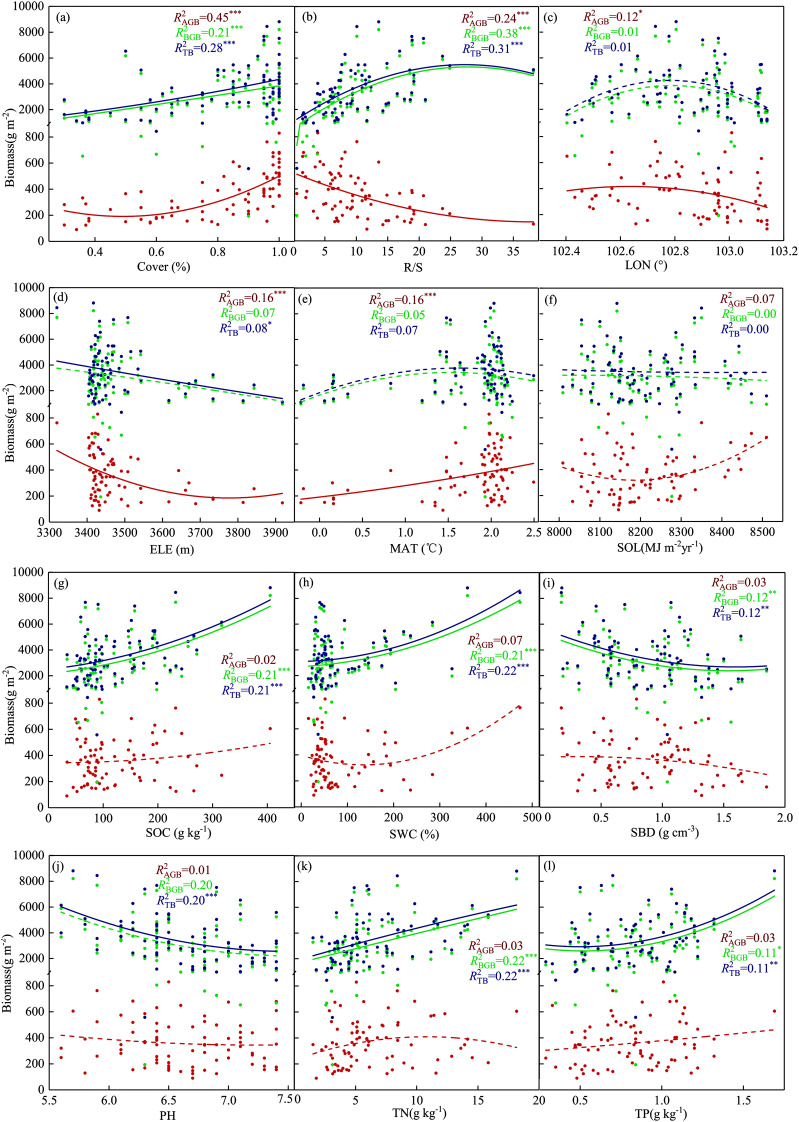
The relationships between 12 key environmental factors (a-l) and aboveground biomass, belowground biomass, and total biomass. The lines represent least squares regression lines, with red, green, and blue points and lines corresponding to aboveground, belowground, and total biomass, respectively. Significance levels are indicated as follows: *P < 0.05; **P < 0.01; ***P < 0.001.

After removing low-relevance indicators from the correlation results, polynomial curve fitting was performed to examine the factors affecting aboveground, belowground, and total biomass (**Figure 4**). Elevation, root-shoot ratio, and longitude were significantly negatively correlated with aboveground biomass (*P*<0.05). Vegetation cover and mean annual temperature were significantly positively correlated with aboveground biomass (*P*<0.001). Solar radiation did not have a statistically significant correlation with aboveground, belowground, and total biomass (**Figure 4f**). Soil bulk density had a significant negative correlation with belowground and total biomass (P<0.01). The root-shoot ratio, vegetation cover, soil organic carbon, soil water content, soil total nitrogen, and soil total phosphorus were significantly positively correlated with belowground and total biomass (P<0.05). In addition to the seven environmental factors that significantly affected belowground and total biomass, elevation and pH were negatively correlated with total biomass (P<0.05).

### 3.4 Relationships between biomass and key factors

**Figure 5:**
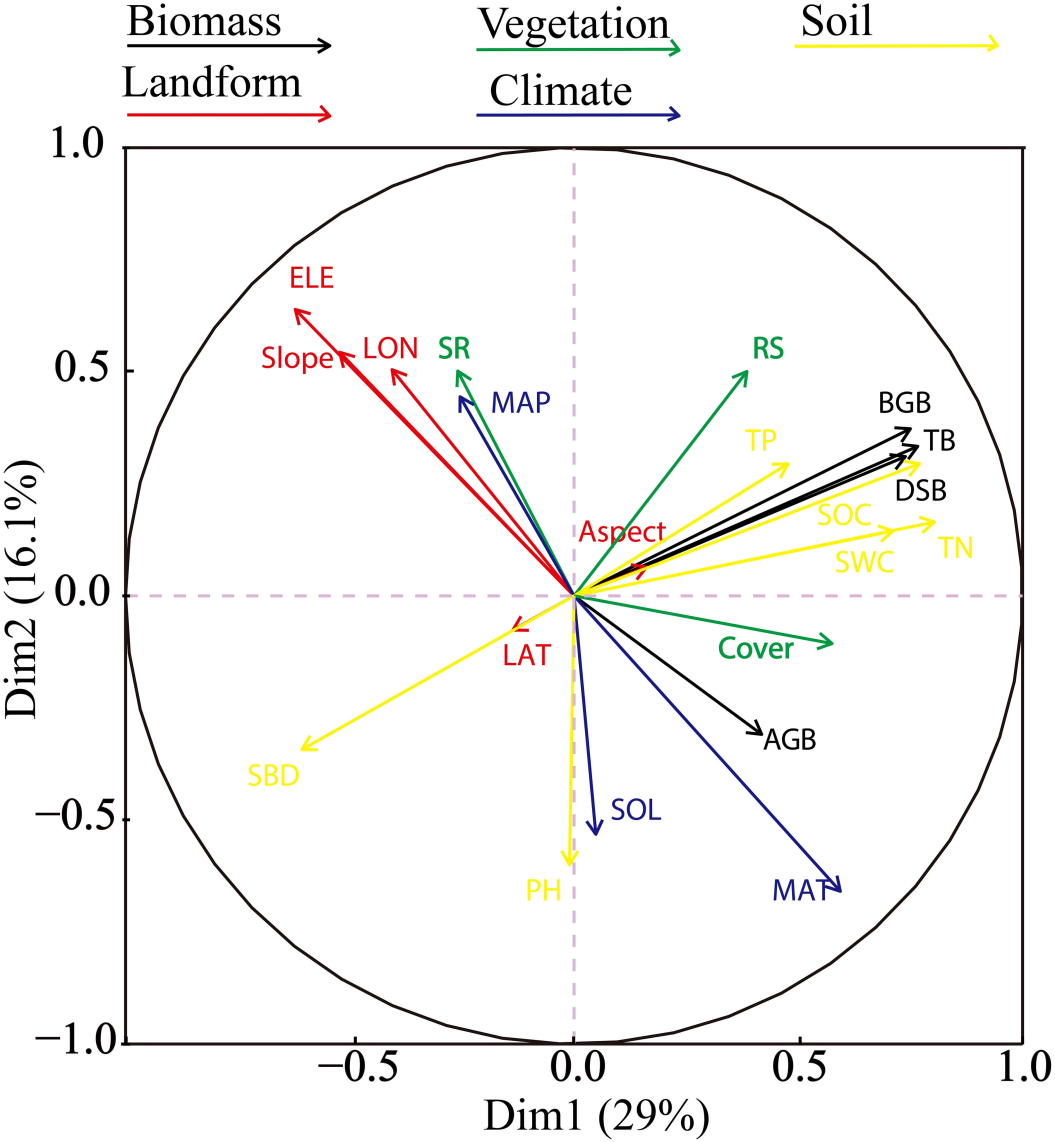
Results of principal component analysis of 18 variables. The arrows represent the eigenvectors corresponding to a variable. The quadrants indicate positive or negative correlations, and the length of the arrow represents the correlation degree.

PCA of 18 variables was used to identify the correlations between the environmental variables and biomass. Two major gradients in the environmental variables were observed, and they explained 45.1% of the variance in the dataset (**Figure 5**). PCA axis 1 primarily reflected the physical and chemical properties of soil, accounting for 29% of the overall variance in the standardized soil variables. This dimension represents a gradient of increasing soil total nitrogen, soil water content, and soil organic carbon. Axis 2 indicated limited contributions of soil chemical properties and primarily reflected the topographic and climate properties. Elevation was negatively correlated with aboveground biomass, whereas belowground and total biomass were positively correlated with soil organic carbon, soil water content, and soil total nitrogen. These parameters tended to decrease with increasing soil bulk density.

The SRA results demonstrate that vegetation cover, root/shoot ratio, elevation, soil water content, and species richness were the key driving factors of aboveground biomass. Vegetation cover, root-shoot ratio, and soil water content had the largest influence on belowground and total biomass. Covariance testing showed that the retained climate, landform, vegetation, and soil variables selected for the SEM exhibited no collinearity (VIF <10) with biomass. The optimal model explained 66.7%, 59.1%, and 56.9% of the variance for aboveground, belowground, and total biomass, respectively.

### 3.5 Multivariate analysis: SEM

**Figure 6:**
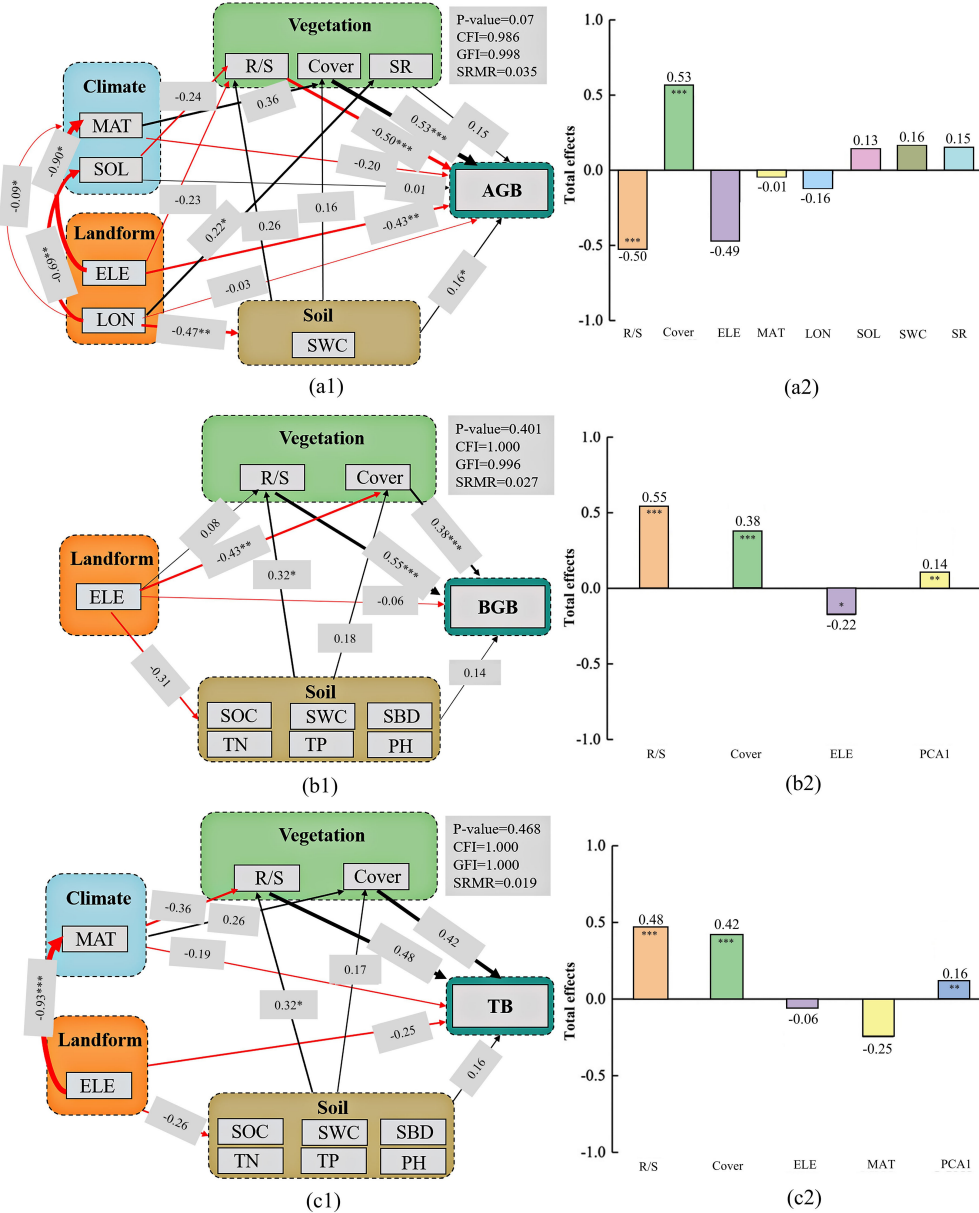
Final structural equation models with environmental factors affecting aboveground biomass **(a1)**, belowground biomass **(b1)**, and total biomass **(c1)**. The path coefficients are for the direct effects, and the total effects comprise direct and indirect effects **(a2, b2, c2)**. The line thickness is proportional to the standardized path coefficients shown next to the line. The significance levels are *P<0.05, **P<0.01, ***P<0.001. Red lines indicate negative correlations and black lines denote positive correlations.

Mean annual temperature, solar radiation, elevation, longitude, soil water content, root-shoot ratio, vegetation cover, and species richness were the critical variables derived from PCA and SRA for modeling aboveground biomass using the SEM (**Figure 6a**). The root-shoot ratio, vegetation cover, elevation, soil organic carbon, pH, soil water content, soil total nitrogen, soil total phosphorus, and soil bulk density were variables in the SEM of belowground biomass (**Figure 6b**). These variables and mean annual temperature were used in the SEM of total biomass (**Figure 6c**). The p-value (>0.05), CFI (>0.95), GFI (>0.95), and SRMR (<0.06) indicated a good model fit. VIFs for AGB, BGB, and TB in the SEM were 1.8, 1.6, and 1.5, respectively. The SEM of belowground and total biomass had six soil indicators. PCA was used for their analysis, and the first principal component was used to represent the soil factors.

The direct effects of vegetation cover, solar radiation, soil water content, and species richness were positive on aboveground biomass, and the path coefficients were 0.53 (*p*<0.001), 0.01, 0.16 (*p*<0.05), and 0.15, respectively. The root-shoot ratio, elevation, longitude, and mean annual temperature had a negative effect on aboveground biomass, and the path coefficients were -0.50 (*p*<0.001), -0.43 (*p*<0.01), -0.03, and -0.20, respectively. Regarding the total effects, the root-shoot ratio (coefficient of -0.50, P<0.001) and vegetation cover (coefficient of 0.53, P<0.001) had a significant effect on aboveground biomass, whereas longitude (coefficient of -0.16) and mean annual temperature (coefficient of -0.01) had a negative but weak effect. However, the root-shoot ratio, vegetation cover, elevation, and Soil PCA1 had significant total effects on belowground biomass (0.55 (P<0.001), 0.38 (P<0.001), -0.22 (P<0.05), and 0.14 (P<0.01), respectively). The patterns of total biomass and belowground biomass were consistent. In addition, the root-shoot ratio and vegetation cover were the dominant factors influencing aboveground biomass, and the soil factors were the leading factors affecting belowground and total biomass. Topographic and climatic factors also had an indirect effect on biomass by affecting plant richness and soil properties, and they also directly affected biomass.

## 4 Discussion

Our study utilized destructive sampling and multivariate modeling to determine the complex interactions between environmental factors and biomass productivity in subalpine wet grasslands. The results provide valuable insights into the effects of different conditions and characteristics on grassland productivity. The relationship between grassland biomass and the dominant factors for an environmental gradient was elucidated. The results revealed abundant belowground carbon storage in the subalpine wet grasslands and different dominant factors affecting aboveground, belowground, and total biomass. In summary, plant traits had a significant effect on aboveground, belowground, and total biomass. Soil factors contributed significantly to belowground and total biomass. Landform and climatic factors had a smaller influence than plant and soil characteristics and impacted biomass directly and indirectly. Plant diversity and soil properties substantially affected the productivity of subalpine grasslands at altitudes of 2000–4000 m.

Although a comprehensive analysis was conducted to assess the impact of environmental factors on grassland biomass, some limitations remained. First, the northeast of the study area is inaccessible, and there were few sampling points, which may have limited the accuracy of biomass estimates in this area. Second, although we had detailed field measurements and determined the correlations between biomass and environmental factors, the limited size of the dataset could have resulted in uncertainties of the SEM. The lack of data in continuous time series is also a constraint. Third, this study focused on the response of subalpine grassland to environmental factors. However, human activities, such as grazing, affected grassland biomass (Hu et al., 2016; Zhou et al., 2017). This study did not assess the impact of human activities on biomass. Other potential variables, such as soil water-holding capacity, soil depth, and growing season length, were excluded due to data availability. The microclimatic variation could be examined in more detail using data other than WorldClim if available. Overall, our results provide insights into the conservation and restoration of grassland ecosystems. Future studies should analyze the contribution of natural and human factors, such as grazing, urban expansion, and other factors, to grassland productivity. It is also worth expanding this dataset to include other subalpine wet grasslands globally.

### 4.1 Biomass estimation

We found that the correlation analysis, quadratic fitting, and stepwise regression models provided different results for the strongest correlation factors. The likely reason is that stepwise regression models can only explain a fraction of the variation in the samples of aboveground, belowground, and total biomass. Although the high variability in biomass parameter values indicates substantial heterogeneity across sites, the grassland biomass we calculated was generally consistent with existing research (Ma et al., 2017). The average belowground biomass was nearly nine times greater than the aboveground biomass, highlighting the importance of the root systems in these ecosystems and demonstrating that belowground biomass is a critical source of soil organic carbon input. Moreover, the high positive correlations between total biomass and belowground and dominant species biomass underscore the critical role of these components in driving productivity. These findings emphasize that roots represent the majority of plant biomass in subalpine wet grasslands, contributing significantly to ecosystem functioning. This is also the reason for the high correlation between belowground and total biomass in subalpine areas.

The root/shoot ratios indicate substantial spatial heterogeneity in biomass allocation. The average root/shoot ratio of 10.10 was significantly higher than that of global temperate grasslands (average of 4.2) (Mokany et al., 2006) and comparable to that of wetland Arctic tundra ecosystems (Jiang et al., 2017). This result is consistent with the significant increase in the root/shoot ratio with declining temperature (Hui and Jackson, 2006). The higher root/shoot ratio in montane areas could be due to the relatively slow depletion of root carbohydrates in response to low respiration rates in cold environments and might be associated with slower root turnover in colder regions (Yang et al., 2009). This elevated root/shoot ratio and high belowground biomass could be a result of adaptation to the severe alpine environment, enhancing stability and nutrient acquisition in challenging conditions (Bardgett et al., 2021; Lewińska et al., 2023).

### 4.2 Environmental factors

Plant biomass in subalpine wet grasslands is closely related to environmental drivers and influenced by abiotic drivers. The positive correlation between vegetation cover and plant biomass detected in this study supports earlier findings of other scholars (Jiang et al., 2017; Zhang et al., 2016). Topographic, meteorological, and soil factors affect the relationship between plant biomass and vegetation cover (Jiang et al., 2017; Liu et al., 2018). The positive correlation between mean annual temperature and biomass contradicts the results of Zhou et al. (2017). The temperature is a limiting factor, and the influence of temperature and solar radiation on biomass is greater than that of precipitation. This may be due to the higher altitude and lower temperatures in the Zoige region (study area) compared to temperate and tropical areas. These conditions inhibit biogeochemical cycles and reduce the availability of soil nitrogen required for plant growth (Yang et al., 2010). Higher altitudes and lower temperatures in montane areas reduce resource effectiveness and the ability of plants to obtain maximum photosynthetic energy, resulting in lower aboveground vegetation biomass at higher altitudes (Shen et al., 2021).

The contents of soil nutrients—such as soil organic carbon, soil total nitrogen, and soil total phosphorus—were positively correlated with both aboveground and belowground plant biomass, albeit with varying sensitivities (see coefficients in **Figure 6**).These nutrients are essential for plants (Hu et al., 2016). Nutrient deficiency slows down plant growth (Merunková and Chytrý, 2012). Soil bulk density was negatively correlated with biomass. High soil bulk density inhibits root growth and infiltration, reducing soil moisture required for plant growth and affecting all soil biochemical processes (Li et al., 2021). Altitude had a large effect on biomass, whereas the correlation with slope and aspect was small. The altitude was negatively correlated with biomass. The likely reason is the lower temperature at higher altitudes, inhibiting plant growth. The shapes of the response curves of the critical environmental factors were highly variable, indicating their spatial variability (Sun et al., 2018).

### 4.3 Multivariate analysis

PCA is a dimensionality reduction technique that transforms a large number of variables into a smaller number of uncorrelated components to explain the majority of the variance in the data (Jolliffe, 2002). This method revealed two key gradients in the environmental variables. The first axis primarily reflected the physical and chemical soil properties, indicating an increase in the contents of soil total nitrogen, water, and organic carbon with an increase in belowground biomass. The second axis, which was related to topography and climate, showed that elevation was negatively correlated with aboveground biomass. In contrast, belowground and total biomass were positively correlated with soil organic carbon, water content, and total nitrogen, and they decreased as the soil bulk density increased. Although PCA can be useful for identifying patterns and reducing dimensionality, it does not model causal relationships or interactions among variables. Therefore, an SEM was developed based on the PCA results to test hypotheses regarding the effects of statistically significant environmental factors.

SEM is a multivariate approach for modeling complex relationships among variables. It provides a comprehensive framework and equations to assess direct, indirect, and total effects (Bollen, 1989) that other multivariate methods, such as PCA, canonical correlation analysis, partial least squares path modeling, and multiple regression, may not fully capture. SEM’s strength is its ability to quantify the direct and indirect effects of factors on the dependent variable, offering insights into the underlying mechanisms of the observed patterns (Kline, 2016). This approach is well-suited for our study, which focuses on disentangling the interactions between multiple environmental factors and their impacts on plant productivity (Grace et al., 2016). However, we acknowledge the potential mathematical dependence of R/S on AGB and BGB. For instance, the VIF values for AGB, BGB, and TB in the SEM ranged between 1 and 2, indicating low multicollinearity. This low-level correlation may arise from the inherent mathematical relationship between R/S and its components, AGB and BGB. Despite this, we retained R/S in the SEM because it represents an ecologically meaningful trait, reflecting biomass allocation strategies across environmental gradients. Given its ecological relevance and the low level of multicollinearity—which is a common and often acceptable issue in SEM—its inclusion is statistically justified.

Therefore, this study used SEM as the core model to investigate the complex relationships between environmental factors and grass biomass in subalpine wet grasslands. The individual influence of plant traits (e.g., root-to-shoot ratio and vegetation cover) on aboveground biomass was greater than that of topographic and soil factors. For belowground biomass, the individual influence of soil factors was greater than that of topographic factors. The root-shoot ratio and vegetation cover had the largest direct and indirect pathway effects. However, topographic and climatic factors had fewer individual effects on aboveground and belowground biomass than soil and plant factors, and the total pathway effect was the lowest, indicating that climate and topographic factors contributed less to plant biomass than soil and vegetation factors. Many studies have shown that climatic factors had the dominant influence on community biomass at large scales (Jiao et al., 2017) and indirectly affected plant community composition by changing soil moisture availability (Yang et al., 2011). In contrast, biodiversity and soil factors affected community density and biomass at small scales (Marquard et al., 2009). Plant traits affect aboveground biomass directly due to resource competition, such as capturing light, whereas soil properties contribute to the decomposition and mineralization of organic matter.

## Data Availability

The raw data supporting the conclusions of this article will be made available by the authors, without undue reservation.
